# Efficacy of green synthesized titanium dioxide nanoparticles in attenuation salt stress in *Glycine max* plants: modulations in metabolic constituents and cell ultrastructure

**DOI:** 10.1186/s12870-025-06194-6

**Published:** 2025-02-18

**Authors:** Reda E. Abdelhameed, Hegazy S. Hegazy, Hanan Abdalla, Marwa H. Adarosy

**Affiliations:** Botany and Microbiology Department, Faculty of Science, Zagazig, University, Zagazig, 44519 Egypt

**Keywords:** Salinity, Nanoparticles, Ultrastructure, Soybean, Antioxidant enzymes, Electrolyte leakage

## Abstract

Salinity is among the major abiotic stresses faced by different countries; limiting plant growth, development and yield. This research work was carried out to evaluate the influence of green prepared titanium dioxide nanoparticles (TiO_2_ NPs) on the growth, metabolic constituents and ultrastructural alterations of soybean (*Glycine max* L.) plants exposed to salt stress. TiO_2_ NPs were green synthesized using an aqueous solution of *Aloe vera* leaf extract and the obtained NPs were identified using several techniques. An in vivo pot experiment was carried out to evaluate the role of foliar sprayed TiO_2_ NPs (30 ppm) on soybean plants irrigated by six NaCl concentrations (0, 25, 50, 100, 150 and 200 mM). After 15 and 30 days from salt application, growth parameters, photosynthetic pigments, total soluble protein, enzymatic antioxidants and ultrastructural changes were tested for potential tolerance of soybean plants growing under salt stress. Results revealed that increasing salt concentrations induced a significant decrease in shoot length, fresh and dry weights as well as the photosynthetic pigments, these decreases were due to increasing electrolyte leakage of soybean plants. However, application of TiO_2_ NPs showed improvements in the vegetative growth by increasing its pigments and protein contents. There was a marked increase in the contents of enzymatic antioxidants in salt stressed soybean plants and further accumulation of their contents with TiO_2_ NPs application. Salt stressed soybean plants showed structural and ultrastructural deformation which was lessened by TiO_2_ NPs application. Finally, our research demonstrates the role of TiO_2_ NPs in alleviating salt stress in soybean plants via restoring the antioxidants and cell ultrastructure, highlighting their potential role as a sustainable and eco-friendly strategy.

**Supplementary Information:**

The online version contains supplementary material available at 10.1186/s12870-025-06194-6.

## Introduction

It is predictable that there will be approximately 10 billion people on the planet by 2050, which indicates a 50% increase in food requirements. The threat posed by the world's population growth and climate change appears to render popular farming approaches obsolete [1, 2]. Nowadays, salinization affects almost one-third of the world's arable land, and it's thought that inadequate irrigation techniques, improper pesticide use, and industrial pollution will make the situation worse on a worldwide scale. Globally, one of the harshest environmental stresses that has a negative impact on the development and biochemical properties of field crops is salinity [3, 4]. Worldwide, plant productivity losses due to salt stress are close to 50% [5]. Severe salts in soils mostly affect osmotic and particular ionic processes, which harm a number of crop plants' metabolic functions such as trigonella [6] and tomato plants [7]. As a result, salt stress negatively impacts plants at every stage of growth, which eventually lowers agricultural yield [8].

One of the main crops affected by different abiotic stresses is soybean (*Glycine max* L.) which is among the significant oil crop with a variety of applications that is becoming more and more well-known globally. It is one of the cheapest and richest supplies of protein for the entire animal sector, particularly to meet the need for protein. On a dry weight basis, one soybean seed has around 40% protein and 21% oil [9]. Soybean root nodules improve soil fertility by fixing nitrogen, in addition to its food and feed qualities. [10]. According to El-Esawi et al. [11], salinity stress negatively impacted the growth and biomass yield, root architecture features, nutrient uptake, chlorophyll, transpiration and photosynthesis rates, soluble proteins and sugars, total phenolics and flavonoid contents of soybean plants. High salinity stress on soybean seedlings has been shown to have negative effects on germination rates and plant growth characteristics. This is because water deficits are known to generate aberrant morphological changes in plants as well as metabolic discrepancies [12, 13].

Therefore, developing a tolerance to salinity is essential for attaining the dual goals of ensuring the world's food security and advancing contemporary agriculture's environmental sustainability. Significant efforts have been made to create salt-tolerant plant genotypes through traditional breeding or genetic engineering to reduce the negative effects of salt stress. However, these attempts have shown limited success, as transgenic plants can easily lose the functional genes responsible for salt tolerance [7, 14]. In order to strengthen, sustain, and increase the output of agricultural systems, it will be necessary to develop new technologies to lessen the challenges caused by salt stress on a global scale [15]. The use of nanotechnology in agriculture has attracted a lot of interest recently [16], because of its capacity to offer quick solutions that are essential to sustainable agriculture [15]. In general, nanoparticles (NPs) are extremely fine matter particles, range in dimension from 1 to 100 nm. According to Agrawal et al. [17], they have the potential to be materials for accomplishing important objectives including enhancing crop development and yield and resilience to adverse stimuli. El-Badri et al. [18] revealed that selenium and zinc oxide NPs modulate the molecular and morpho-physiological processes during seed germination of *Brassica napus* under salt stress. Among the other different types of NPs, titanium dioxide (TiO_2_) NPs are widely employed due to their unique properties, including their small size, high surface area, their chemical stability and non-toxic nature [19]. Their effects on plants may be beneficial or detrimental depending on the concentration, method of application, plant species and environmental conditions [20]. TiO_2_ NPs have been shown to have a number of beneficial and positive effects on the morphophysiological and biochemical characteristics of different agricultural plants [21–23]. Alharby et al. [24] showed that the growth, chlorophyll contents, and nutritional content of wheat plants were all boosted by TiO_2_ NPs. Some other studies have found detrimental effects of TiO_2_ NPs on plant growth (e.g., reduced cell elongation, reduced transpiration and leaf growth) [25, 26].

Modern physical and chemical methods employ a variety of techniques to synthesize NPs which often involve toxic reagents, high energy consumption and hazardous by-products. However, the development of successful green synthesis techniques has drawn researchers to biological techniques, which use plant extracts as an easy, affordable, environmentally beneficial and non-toxic substitute [27]. The utilization of *Aloe vera* for the NPs synthesis enhances their biological activity. Moreover, it enables regulation of the size and shape of the synthesized NPs, which can enhance their therapeutic potential. *A. vera* plant includes a huge variety of vital substances, including terpenoids, flavonols, and phenols, which function as reducers and stabilizers for the formation of NPs [28]. Taken the advantages of green synthesis of TiO_2_ NPs using *A. vera* aqueous extract [29], this study was aimed to use green, eco-friendly synthesized TiO_2_ NPs to alleviate the negative effects of salinity on soybean plants via investigating growth, metabolic constituents and cell ultrastructure.

## Materials and methods

### Chemicals

All the chemicals were purchased from commercial sources such as Sigma-Aldrich. All chemicals were of analytical grade were used as-received without any further purification.

### Green synthesis of TiO_2_ NPs from *A. vera* L. extract

In accordance with Hanafy et al. [30], 1000 mL of distilled water was combined with 250 g of the *A. vera* leaves, and the mixture was heated for two hours at 90 °C. Following cooling, Whatman No. 1 filter paper was used to filter the extract in order to purify it. After that, the extract was kept at -4 °C to facilitate the subsequent production of TiO_2_ NPs using titanium tetrachloride (TiCl_4_) as a precursor. Briefly, 100 mL of *A. vera* leaves extract was added dropwise to a 100 mL of 1.0 N TiCl_4_ solution in deionized water. The pH value was adjusted to 9 then the obtained suspension was filtered to separate the formed NPs. Several characterization techniques, including X-ray diffraction (XRD), Fourier transform infrared (FTIR), transmission electron microscopy (TEM), scanning electron microscopy (SEM), and ultraviolet spectrophotometer, were used to characterize TiO_2_ NPs. The synthesis and characterization of TiO_2_ NPs were done through a study of Abdalla et al*.* [29].

TiO_2_ NPs were suspended at a concentration of 30 ppm in deionized, distilled water. To guarantee that these NPs were distributed and to prevent agglomeration and aggregation, the suspensions were sonicated for four hours in a bath sonicator (Branson's Model B200 ultrasonic).

### Application of green synthesized TiO_2_ NPs on soybean seeds

#### In vivo* greenhouse study (pot experiment)*

Soil was collected from Sharqia Governorate and the main chemical and physical characteristics were determined in soil–water extract (1:5 w/v) according to Jackson [31] in the central lab., Faculty of Agriculture, Mansoura University. Using the hydrometer approach, the particle size distribution (clay, silt, and sand) was determined. The ammonium acetate method [32] was used to measure exchangeable cations, and the electrometric method [33] was used to determine the pH of the soil (1:1 soil: water ratio). The available concentrations of K^+^, Ca^2+^, Na^+^ and Mg^2+^ by atomic absorption spectrophotometer (model unicam 969) were determined according to Nation and Robinson [34] as recorded in Table 1.

**Table 1:** Physical and chemical characters of soil used in this study

Soil property	Value	
**Soil texture**	**62.82**	**Clay %**
	**18.33**	**Silt %**
	**18.85**	**Sand %**
**Physical characteristics**	**7.64**	**pH**
	**1.15**	**EC(dS m**^**−1**^**)**
	**54.57**	**Saturation percent (%)**
	**27.29**	**Field capacity**
**Chemical characteristics**	**3.18**	**SO**_**4**_^**−2**^
	**2.1**	**Cl**^**−**^
Anions (meq/100 g soil)	**0.37**	**(HCO**_**3**_**)**^**−**^
	**0.05**	**(CO**_**3**_**)**^**−**^
Cations (meq/100 g soil)	**1.45**	**Mg**^**+2**^
	**2.19**	**Ca**^**+2**^
	**0.35**	**K**^**+**^
	**0.8**	**Na**^**+**^

Soybean seeds (*G. max* L. var. 35) were acquired from the Food and Legumes Research Department, Field Crops Research Institute, Agricultural Research Centre, Giza, Egypt during March, 2022. A soybean pot experiment was conducted at the greenhouse of Botany and Microbiology Department, Faculty of Science at Zagazig University. About 2 kg of soil was taken in plastic pot of 24 cm diameter and supplied with tap water. In the following day, for ten minutes, seeds were surface sterilized using 0.5% sodium hypochlorite then 10 seeds were planted in each pot. Plants were grown under greenhouse condition (light/dark cycle: 25 ± 2ºC light period, 20 ± 2ºC dark). Young seedlings were irrigated with only fresh water throughout the first few weeks of the plantation. After that, each container was stocked with five plants in order to reach the proper density.

#### *NaCl and TiO*_*2*_*NPs treatment*

The experiment was done using six varying concentrations of NaCl (0, 25, 50, 100, 150 and 200 mM) and two TiO_2_ NPs concentrations (0 and 30 ppm) with a total 12 (6*2) treatments, and each treatment was replicated 3 times (36 pots). After two weeks from sowing, NaCl solutions were added gradually and irrigations were performed at sunset, two times a week by different salt concentrations. In a regular with salt irrigation, a constant volume of TiO_2_ NPs at 30 ppm concentration was sprayed twice a week by a hand pump sprayer. Non treated plants were used as control and were irrigated and sprayed with water.

#### Plant sampling and analysis

At intervals of 2 weeks, when the effects of salt and TiO_2_ NPs were appeared on the plants, both control and treated plant samples were collected at fixed time in 2 periods (15 and 30 days after treatment with salt). Part of these samples is used for measuring growth parameters, another part was liquid nitrogen-frozen and kept at -20 °C for physiological and biochemical analysis. As well, structural and ultrastructural alterations were examined after 30 days of salt application.

#### Morphological and growth measurements

Plant growth measurements including shoot height (cm), fresh and dry weights (g) of both shoot and root were recorded after 15 and 30 days from salt application. To test the aforementioned features, a random sample of three plants was taken from each treatment. Three samples were weighed separately for both the fresh and dry weights. The samples were then maintained in the oven for 72 h at 60 °C to measure the dry weight.

## Physiological investigation

### Quantitative estimation of total chlorophyll and pigments

Pigment concentration was determined based on the procedure of Metzner et al. [35]. Soybean leaves were ground in a mortar using 5 mL of 85% cold aqueous acetone solution with pre-washed sand. After centrifuging the homogenate, 85% acetone was added to the supernatant to bring it up to a fixed volume of 10 mL. In comparison to a blank of 85% acetone, the optical density was assessed at 663, 644, 452.5 nm using spectrophotometer. Total chlorophyll (Chl. a + Chl. b) and total pigments (Chl. a + Chl. b + carotenoids) were estimated and stated as mg/g.

### Plasma membrane permeability and membrane stability index (MSI)

Plasma membrane permeability was measured in line with the approach of Shi et al. [36] in term of electrolyte leakage (EL). In order to eliminate the electrolytes generated during leaf disc excision, ten leaf discs from the young, leaves were placed in 50 mL glass vials and rinsed with distilled water. After that, vials containing 30 mL of distilled water were placed in the dark and left to remain at room temperature for 24 h. Using a conductivity meter, the solution's first conductivity (EC1) was determined at the end of the incubation period. After 15 min of heating at 95ºC in a water bath, the vials were allowed to cool to room temperature before the EC2 was measured. By Farooq and Azam [37], the membrane stability index (MSI) was estimated. The relative EL and MSI were computed using the subsequent equation.

$$\text{EL}\%=\frac{\text{EC}1}{\text{EC}2}\times 100$$

$$\text{MSI}=\left[1-\frac{\text{EC}1}{\text{EC}2}\right]\times 100$$

### Biochemical analysis

#### Analysis of the total soluble protein content

Using bovine serum albumen as a reference, the total soluble protein was calculated using the Lowry et al. [38] method. Its concentration was then represented as µg/g fwt.

### Investigation of enzymatic antioxidant

## Extraction

Plant samples were extracted with 5 mL of ice cold 50 mM potassium phosphate buffer (pH 7.0) containing 1 mM Ethylene Diamine Tetra Acetic acid. In the event of an ascorbate peroxidase (APX) test, 5 mM of ascorbic acid were added. For 10 min, the homogenate was centrifuged at 8000 rpm and 4 °C. Antioxidant enzyme assays were conducted using this supernatant.

### Activity of peroxidase (POX)

With slight modifications, the activity of POX was assessed using the methodology of Chance and Maehly [39]. The reaction mixture consisted of 0.33 mM pyrogallol, 10 mM potassium phosphate buffer (pH 7.0), and 0.5 mL of enzyme extract. The reaction was initiated by adding 40 mM H_2_O_2_. As purpurogallin formed in the presence of H_2_O_2_, an increase in absorbance at 470 nm was observed, which allowed for the determination of the activity. The POX activity was reported and calculated using the following equestion.

$$\text{Units}\,(U)/g\,\text{fwt}=\frac{\{A470nm(test)-A470nm (blank)\}\ast TV\ast DF}{\upepsilon 470\ast \text{VU}\ast\text{fwt}}\times\,100$$

A_470nm_ = Absorbance at wavelength 470 nm.

TV = Total volume (in milliliters) of reaction.

DF = Dilution factor of enzyme.

ε_470_ = Molar extinction coefficient A_470 nm_.

VU = Volume used (in milliliters) of enzyme used.

Fwt = fresh weight of sample.

### Activity of catalase (CAT)

Activity of CAT was measured along with Aebi [40], with some modifications. The reaction was initiated by adding 0.5 mL of enzyme extract to 3 mL of reaction mixture containing 50 mM potassium phosphate buffer (pH 7.0) and 10 mM H_2_O_2_. CAT activity was determined as the consumption of H_2_O_2_ at 240 nm. Activity of CAT was stated using extinction coefficient (ε_240_ = 43.6 M^−1^ cm^−1^).

### Activity of APX

Nakano and Asada [41] state that APX activity was carried out using a reaction mixture containing 5 mM H_2_O_2_, 0.25 mM ascorbic acid, and 50 mM phosphate buffer (pH 7.0) and 125 µL of enzyme extract, By monitoring ascorbic acid oxidation in the presence of H_2_O_2_, the activity was ascertained by measuring the absorbance at 290 nm, then the APX activity was estimated using extinction coefficient (ε_290_ = 2.8 mM^−1^ cm^−1^).

### Structural and ultrastructural examination

Leaf samples from four different treatments (control, TiO_2_ NPs, salt (150 mM) and TiO_2_ NPs + salt (150 mM)) were taken after 30 days of salt application and administered for transmission electron microscopy (TEM) by the technique designated by Perera and Gay [42]. The plant leaf was cut into pieces and fixed for 24 h at room temperature with 2.5% glutaraldehyde in a 0.1 M sodium cacodylate buffer at pH 7.0. The specimens were then washed three times in fresh buffer solution and post-fixed for an hour in 1% (v/v) osmium tetroxide in 0.1 M sodium cacodylate buffer. Following post-fixation, the tissues were dehydrated in a series of alcohol.

The leaf pieces were then soaked in epoxy propane-absolute ethanol solution (50:50) and left in closed containers for 30 min, then they were transferred into pure epoxy propane (100%) and left for another 30 min. The pure epoxy propane was then replaced by a mixture of equal parts of epoxy propane and Spurs resin (1:1) and left for an hour in closed containers (embryo cups). The lids of the embryo cups were left slightly open, allowing epoxy propane to evaporate overnight. Then, after several changes in fresh resin over 2–3 days, tissue fragments were embedded in fresh resin in embedding capsules with the specimen number written in pencil on a small slip of paper, placed in the center of the capsule, and left in an oven at 60ºC to polymerize.

### Examination on light microscope

Semithin Sects. (1 µm thick) were cut using glass knives and the sections floated on water surface were picked up using eye lash and placed on a drop of water on a glass slide. The slide was then heated gently to get rid of the water drop and the sections were adhered to the slide. A drop of toluidine blue stain was placed on the sections for 90 s, then washed by distilled water and the sections were examined by light microscope.

### Examination on TEM

Ultra-thin sections were cut by ultramicrotome. Silver or gold sections were picked up on a dull surface of formvar (polyvinyl formaldehyde) coated copper grids. A piece of filter paper was slipped between the tips of a pair of forceps to remove the film. Ultra-thin sections were double stained on a wax plate placed in a Petridish. Pellets of sodium hydroxide were placed in the Petridish to remove carbon dioxide from the environment. A drop of 2% aqueous uranyl acetate [43] was pipetted on the wax plate and the mounted grids were gently floated with the sections facing down on the drop of the stain. The dish was immediately covered with a lid. The sections were left for 30 min in uranyl acetate and then washed in a gentle stream of glass distilled water and dried on filter paper. The same method was adopted for lead citrate staining, the sections being placed in Reynold's lead citrate solution [44] and left for 10–20 min, then washed and dried for uranyl acetate staining. Stained sections were examined with TEM (JEOL JEM-2100) at Electron Microscope Unit, Faculty of Agriculture, Mansoura University.

### Statistical analysis

The tables and graphs display the means ± standard errors based on data from three replicas (*n* = 3). ANOVA, or analysis of variance, was employed to statistically validate the results and the computations were done using SPSS® 15.0. To ascertain whether there was a significant difference between the control and treatment groups, the Duncan's multiple range test (*p* ≤ 0.05) was employed. Data inter-relationship between different treatments [principal component analysis (PCA) and Hierarchical clustering analysis (HCA)] were performed using Past program for scientific data analysis.

## Results

TiO_2_ NPs were made utilizing a green method that involved the use of liquid extract from *A. vera* leaves.. The observed change in color (pinkish brown color from milky) of titanium solution was considered as a confirmation of the reduction of TiCl_4_ salt into TiO_2_ NPs [30]. The spectral analysis of the change in this color gave a particular peak between 200 and 300 nm. Surface, size and the particle morphology of TiO_2_ NPs were imaged by SEM and TEM. According to the results of the SEM study, TiO_2_ NPs have a tetragonal structure, and the majority of the nanoforms have sizes between 10 and 25 nm. To examine the sample's structure and phase development, TiO_2_ NPs' FTIR and XRD were examined. TiO_2_ NPs showed a well-crystallized anatase profile based on XRD, which verified the tetragonal structure of the material. These results were all recently published in our earlier paper [29]. Here, in this study we concentrated on the application of these green prepared TiO_2_ NPs on mitigation the salinity detrimental effects on soybean plants.

### Application of green synthesized TiO_2_ NPs on soybean plants under salt stress

#### Growth response

The results observed in Suppl. Figure 1 and Table 2 showed that varying levels of salt stress adversly affect the growth responses of soybean plants (shoot height, fresh and dry weight of shoot and root). The data of shoot height of soybean is shown in Table 2, where there was a gradual decrease in shoot height with increasing NaCl concentrations. The shoot height decreased by 3.84 and 26.9% at the lowest (25 mM NaCl) and the highest (150 mM NaCl) salt concentration, respectively after 30 days compared with control. Most obviously that with TiO_2_ NPs spraying, salt treated plants showed less decrease in shoot height by 1.92 and 11.53% at 25 and 150 mM NaCl relative to control after 30 days from salt treatment. TiO_2_ NPs decreased the harmful effect of salinity, where considerably amplified shoot heights of soybean plants grown in control and salinized soil during all growth periods.

**Table 2:** Shoot heights (cm), shoot and root fresh (fwt) and dry weights (dwt) (g/plant) of TiO_2_ NPs (+ NPs) and non-TiO_2_ NPs (-NPs) treated soybean plants grown under different NaCl concentrations

Treatments		Shoot height		Shoot fwt		Root fwt		Shoot dwt		Root dwt	
**NaCl conc. (mM)**	**NPs (30 ppm)**	**15**	**30**	**15**	**30**	**15**	**30**	**15**	**30**	**15**	**30**
**0**	**-NPs**	23.5 ± 0.65b	26 ± 0.687b	2.8 ± 0.07ab	2.96 ± 0.08b	0.75 ± 0.02bc	1.3 ± 0.03ab	0.4 ± 0.009b	0.45 ± 0.01c	0.15 ± 0.003a	0.17 ± 0.004d
	** + NPs**	25 ± 0.66a	27 ± 0.714a	3.35 ± 0.09a	3.8 ± 0.098a	0.82 ± 0.021a	1.28 ± 0.03a	0.43 ± 0.01a	0.54 ± 0.014a	0.16 ± 0.004a	0.22 ± 0.005a
**25**	**-NPs**	18.5 ± 0.5de	26 ± 0.687b	2.76 ± 0.1bc	2.8 ± 0.07bc	0.65 ± 0.017d	1.2 ± 0.03bc	0.3 ± 0.008d	0.45 ± 0.011c	0.136 ± 0.003b	0.145 ± 0.003e
	** + NPs**	21.5 ± 0.6bc	25 ± 0.661c	3.27 ± 0.09a	3.66 ± 0.1a	0.79 ± 0.02a	1.2 ± 0.03ab	0.34 ± 0.009c	0.53 ± 0.013a	0.15 ± 0.003a	0.21 ± 0.005b
**50**	**- NPs**	17.5 ± 0.46e	23.5 ± 0.621e	2.6 ± 0.069c	2.72 ± 0.07c	0.51 ± 0.013e	1.07 ± 0.03d	0.31 ± 0.007d	0.44 ± 0.008d	0.13 ± 0.003b	0.14 ± 0.003e
	** + NPs**	21 ± 0.582c	24.5 ± 0.65cd	2.94 ± 0.08b	3.6 ± 0.095a	0.76 ± 0.02bc	1.1 ± 0.03cd	0.32 ± 0.008d	0.506 ± 0.006b	0.14 ± 0.003b	0.19 ± 0.004c
**100**	**- NPs**	16.5 ± 0.43f	20 ± 0.529f	2.61 ± 0.07c	2.7 ± 0.07c	0.44 ± 0.011f	0.95 ± 0.025e	0.28 ± 0.007d	0.434 ± 0.011c	0.11 ± 0.002d	0.13 ± 0.003e
	** + NPs**	19.5 ± 0.52d	24 ± 0.634d	2.69 ± 0.07c	3.5 ± 0.092a	0.75 ± 0.02bc	0.97 ± 0.025e	0.29 ± 0.007d	0.48 ± 0.006d	0.12 ± 0.003c	0.14 ± 0.003e
**150**	**-NPs**	15.5 ± 0.4fg	19 ± 0.502fg	1.64 ± 0.04e	1.87 ± 0.05e	0.35 ± 0.009g	0.9 ± 0.022f	0.2 ± 0.005f	0.4 ± 0.06e	0.1 ± 0.002e	0.08 ± 0.002f
	** + NPs**	16.6 ± 0.44f	22 ± 0.608e	1.95 ± 0.05d	2.4 ± 0.06d	0.72 ± 0.018c	0.78 ± 0.02f	0.3 ± 0.006e	0.5 ± 0.008d	0.11 ± 0.002e	0.14 ± 0.003e
**200**	**- NPs**	12 ± 0.317h	-	1.17 ± 0.03f	-	0.33 ± 0.008g	-	0.1 ± 0.002g	-	0.06 ± 0.001f	-
	** + NPs**	13 ± 0.343g	18.5 ± 0.57g	1.24 ± 0.03f	1.6 ± 0.04f	0.36 ± 0.009g	0.7 ± 0.017g	0.2 ± 0.005f	0.4 ± 0.012c	0.09 ± 0.002e	0.11 ± 0.002f

^*^Data represent means ± standard errors of three biological replicates. Within each column for each parameter, different letters (a, b, c, d…) indicate significant difference (*p* < 0·05), according to a Duncan multiple range test

Concerning the fresh and dry weights of shoots and roots, salinity stress significantly reduced these parameters compared with the control treatment as showed in Table 2. Also, there was a gradual decline in these parameters with elevating the soil's salinity content. Nevertheless, there was highly remarked decrease in non-TiO_2_ NPs than TiO_2_ NPs sprayed plants, where TiO_2_ NPs mostly improved fresh and dry matter in the salt stressed plants. The percent decrease in root and shoot dry weights of TiO_2_ NPs soybean plants at the 150 mM salt level was 19.27 and 2.17%, respectively after 30 days, over non- TiO_2_ NPs ones. Most obviously that soybean plants treated with 200 mM NaCl were unable to tolerate the high salinity and died after 30 days of salt application.

### Plasma membrane permeability and MSI

**Fig. 1:**
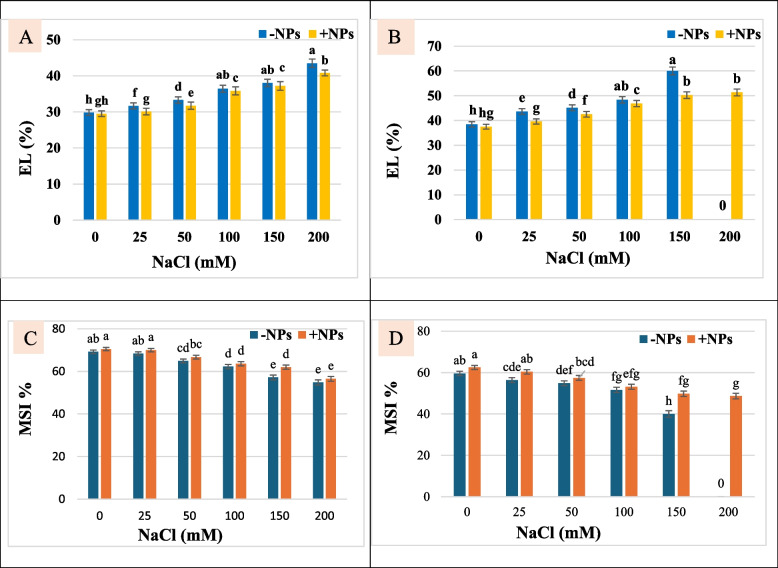
Electrolyte leakage (EL) and membrane stability index (MSI) of TiO_2_ NPs (**+ **NPs) and non-TiO_2_ NPs (**-**NPs) treated soybean plants grown under different NaCl concentrations after 15 days (**A** and **C**) and 30 days (**B** and **D**) of salt application. * Data represent means ± standard errors (error bars) of three biological replicates. Different letters above column (a, b, c, d…) indicate significant difference (*p* < 0·05), according to a Duncan multiple range

Plasma membrane permeability or EL of soybean plants increased with increasing NaCl concentration and with time (Fig. 1A, B). Spraying with TiO_2_ NPs reduced EL of soybean plant leaves when compared with non-TiO_2_ NPs plants grown under control or salt stessed conditions. An increase of 18.39 and 25.83 in EL at 50 and 100 mM NaCl was observed relative to control one. However, TiO_2_ NPs treated plants showed less decrease in EL indicated that reduction in EL of soybean plants leaves was more pronounced in TiO_2_ NPs spraying plants. Data drawn in Fig. (1C, D) presented the enhancement effect of TiO_2_ NPs on soybean plants by increasing MSI relative to non-TiO_2_ NPs applied plants.

#### Pigment fractions

The results in Table 3 showed the impact of various salinity levels and TiO_2_ NPs application on photosynthetic pigment contents. These findings demonstrated that whereas total chlorophyll (Chl a and b) and total pigments (Chl a, b and carotenoids) dropped as salt concentrations increased, soybean plants treated with TiO_2_ NPs shown a lessened decrease. At all salinity levels, the photosynthetic pigment concentrations of TiO_2_ NPs plant leaves were generally much higher than those of non-TiO_2_ NPs plant leaves. Most obviously that the concentrations of total chlorophyll and total pigments were 1.92 and 2.198 mg/g fwt respectively at 100 mM NaCl stress in non-TiO_2_ NPs plants after 15 days. While, treatment with TiO_2_ NPs caused an enhancement in these content recording, 2.11 and 3.04 mg/g fwt respectively at 100 mM NaCl stress after 15 days.

**Table 3:** Total chlorophyll and total pigments (mg/g fwt) of TiO_2_ NPs (+ NPs) and non-TiO_2_ NPs (**-**NPs) treated soybean plants grown under different NaCl concentrations

Treatments		Total chlorophyll		Total pigments	
**NaCl conc. (mM)**	**NPs (30 ppm)**	**Days after salt treatment**			
		**15**	**30**	**15**	**30**
**0**	**-NPs**	2.54 ± 0.07b	2.84 ± 0.024b	3.23 ± 0.11b	3.58 ± 0.045b
	** + NPs**	2.9 ± 0.073a	3.26 ± 0.029a	3.92 ± 0.106a	4.36 ± 0.038a
**25**	**-NPs**	2.49 ± 0.023d	1.74 ± 0.045d	3.028 ± 0.038bc	2.407 ± 0.07d
	** + NPs**	2.55 ± 0.022b	1.94 ± 0.052c	3.57 ± 0.044c	2.70 ± 0.08c
**50**	**-NPs**	2.021 ± 0.016f	1.63 ± 0.043e	2.968 ± 0.033e	2.002 ± 0.07e
	** + NPs**	2.35 ± 0.017c	1.752 ± 0.051cd	3.304 ± 0.034c	2.57 ± 0.068bc
**100**	**-NPs**	1.82 ± 0.011fg	1.39 ± 0.018g	2.198 ± 0.028f	1.89 ± 0.030d
	** + NPs**	2.11 ± 0.013e	1.65 ± 0.045de	3.04 ± 0.022d	2.485 ± 0.065d
**150**	**-NPs**	1.63 ± 0.007h	1.093 ± 0.037f	1.947 ± 0.02h	1.6 ± 0.05de
	** + NPs**	1.97 ± 0.009g	1.433 ± 0.027e	2.804 ± 0.026e	2.032 ± 0.041de
**200**	**-NPs**	0.43 ± 0.012j	-	0.842 ± 0.019f	-
	** + NPs**	1.47 ± 0.007i	0.766 ± 0.023h	2.09 ± 0.045g	1.117 ± 0.038e

^*^Data represent means ± standard errors of three biological replicates. Within each column for each parameter, different letters (a, b, c, d…) indicate significant difference (*p* < 0·05), according to a Duncan multiple range test

#### Total soluble protein content

**Fig. 2:**
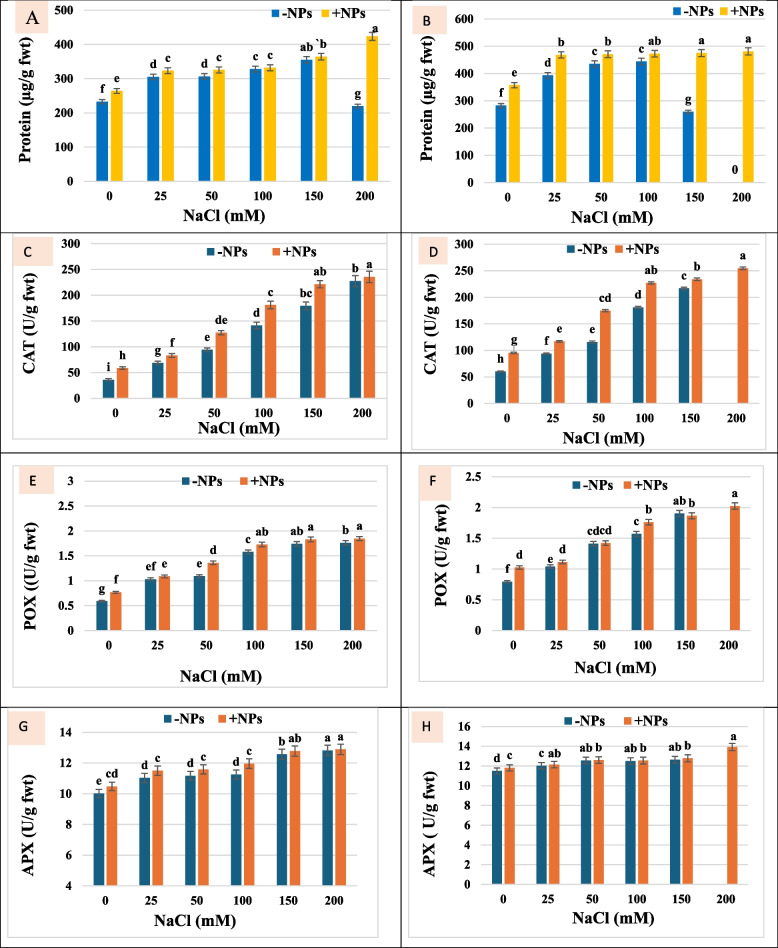
Protein content (µg/g fwt) and antioxidant enzymes (CAT: catalase, POX: peroxidase and APX: ascorbate peroxidase) of TiO_2_ NPs (+ NPs) and non-TiO_2_ NPs (**-**NPs) soybean plants grown under different NaCl concentrations after 15 days (**A**, **C**, **E** and **G**) and 30 days (**B**, **D**, **F** and **H**) of salt application. * Data represent means ± standard errors (error bars) of three biological replicates. Different letters above columns (a, b, c, d…) indicate significant difference (*p* < 0·05), according to a Duncan multiple range test

Generally, salt stress increased the total soluble protein contents in soybean plant leaves as compared with control (Fig. 2A, B), and a further upsarge in their contents were significantly observed after TiO_2_ NPs usage under saline and non-saline conditions, in comparison to their respective control. At the lowest NaCl level (25 mM), the soluble protein content was 305.2 µg/g fwt after 15 days, where its content was 393.15 µg/g fwt after 30 days of salt application. On the other hand, protein content increased greatly with TiO_2_ NPs recording 475.05 and 481.15 µg/g fwt at 150 and 200 mM NaCl respectively after 30 days.

#### Antioxidant enzymes

**Fig. 3:**
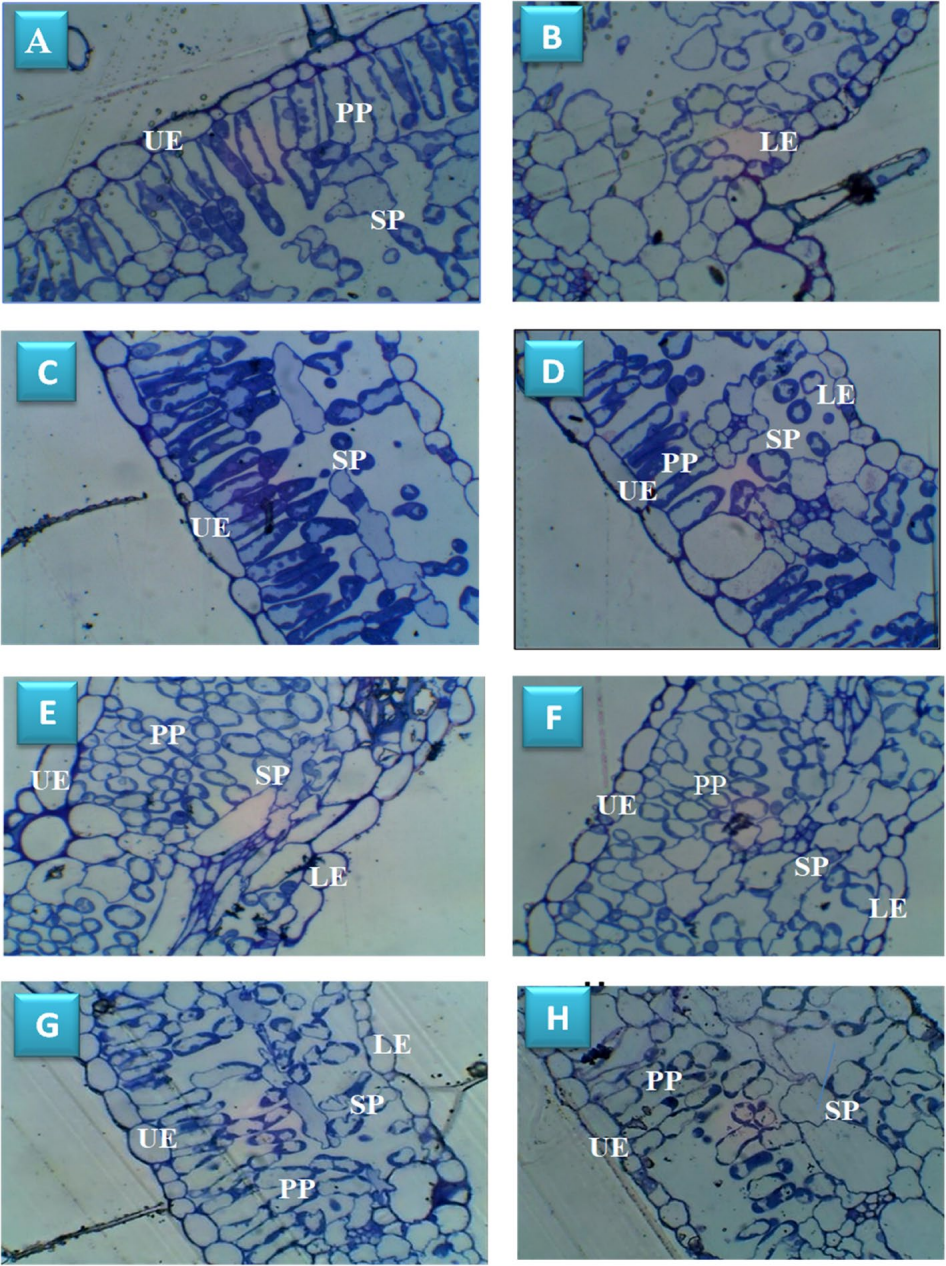
Light microscopy photographs showing transverse sections of soybean leaf tissue**;** (**A**, **B**) untreated control plant; (**C**, **D**) Plant treated with TiO_2_ NPs; (**E**, **F**) 150 mM NaCl treated plant**; (G, H)** 150mM NaCl treated plant and sprayed with TiO_2_ NPs. **UE**—upper epidermis, **LE**— lower epidermis, **PP**—palisade parenchyma, **SP**—spongy parenchyma

This study looked at the activity of three antioxidant enzymes in soybean plants: APX, POX and CAT in an attemp to determine whether the enzymatic system could be utilised to assess whether the application of TiO_2_ NPs induced salt tolerance by scavenging ROS. Salt stress resulted in general increment in the antioxidant enzyme of soybean plant shoots. Most interestingly that spraying with TiO_2_ NPs caused further enhancement in the antioxidant enzymes as compared to non-TiO_2_ NPs applied ones (Fig. 3C-H). Under control condition, TiO_2_ NPs application increased CAT, POX and APX by 58.57, 29.13, and 2.69%; respectively. Whereas, under different salt concentrations, these values were significantly augmented.

#### Structural examination

Light microscopy studies on leaves of both control and TiO_2_ NPs treated soybean plant (Fig. 3 A-D) showed normal and regular palisade and spongy layers. In TiO_2_ NPs treated soybean plant, more number of chloroplasts in the palisade layer were observed which attached regularly to the cell membrane compared with control (Fig. 3 C, D). In leaves of 150 mM treated soybean plant, palisade parenchyma cells are irregular with low chloroplast content as well as an increase in the area of intercellular spaces were observed (Fig. 3 E, F). Clearly, the application of TiO_2_ NPs in salt stressed soybean revealed a modulation in cell structure by maintaining, to some extent, the shape of both palisade and spongy layers compared with salt stressed plant only (Fig. 3 G, H).

#### Ultrastructural examination

**Fig. 4:**
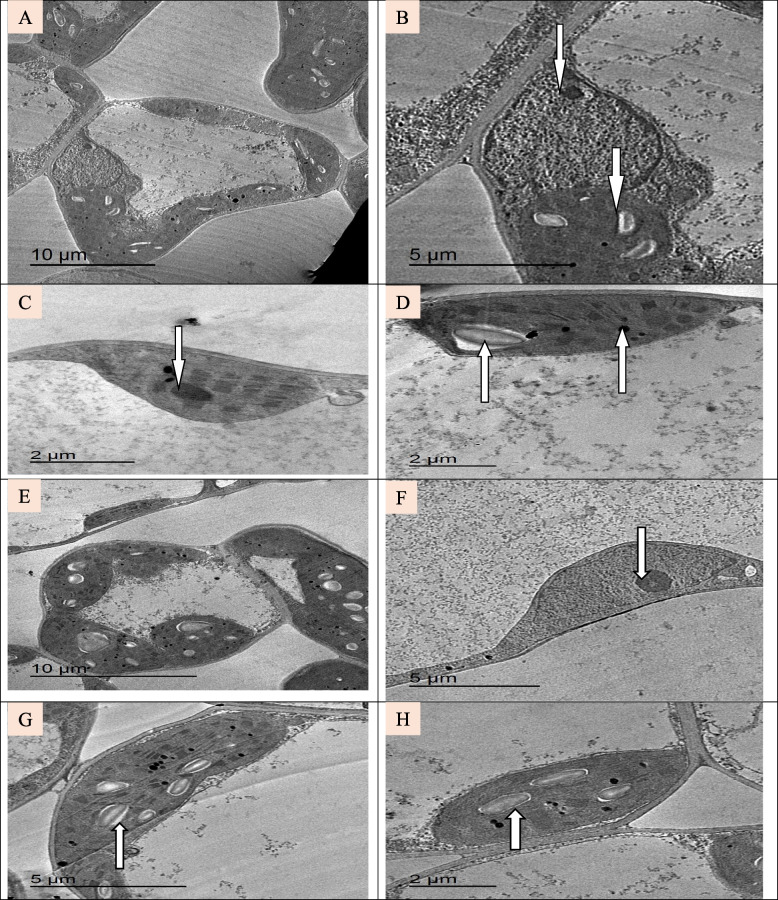
(**A-D**):Transmission electron micrographs of leaf tissue of control un-sprayed with TiO_2_ NPs soybean plant; (**A**, **B**) Normal cell structure; (**C**, **D**) Normal appearance of chloroplast, (**E–H**): Transmission electron micrographs of leaf tissue of soybean plant sprayed with TiO_2_ NPs; (**E**, **F**) Normal cell structure has normal nucleus with defined nucleolus and complete nuclear membrane; (**G**, **H**) A large chloroplast containing many starch grains and clear structure of grana, the arrows refer to normal nucleus, starch grains and osmiophilic lipid granules/plastoglobuli

To better understand the effects of the applied TiO_2_ NPs at subcellular levels, the leaves of soybean were analyzed through transmission electron microscopy (TEM). A typical ultrastructure was exhibited in control and TiO_2_ NPs treated cells (Fig. 4) The cell wall appeared thick and normal. Plasma membrane was unfolded with a uniform shape in all parts lying close to the cell wall (Fig. 4 A, B). The organelles were immersed in cytoplasm. The nucleus was observed with distinct nucleolus and enclosed by a well defined double nuclear membrane (Fig. 4 B). The detailed structure of chloroplast, grana and stroma lamellae are normal (Fig. 4 C, D). Chloroplast is with normal appearance of starch grains and intact membrane and present in high number in each cell. In the plants treated with TiO_2_ NPs, chloroplast is bigger and has a large number of starch grains (Fig. 4 E–H).

**Fig. 5:**
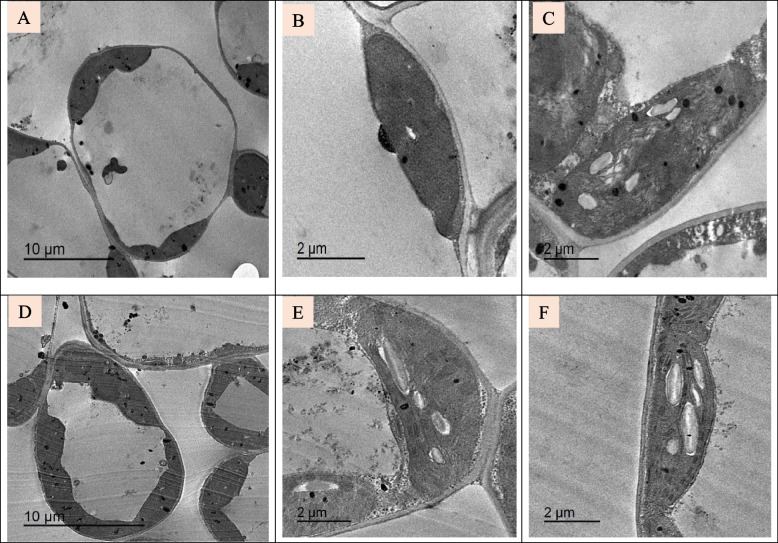
(**A-C**): Transmission electron micrographs of leaf tissue of soybean treated with 150 mM NaCl; (**A**) The cell contains small number and size of chloroplasts; (**B** and **C**) Disorganized chloroplasts. (**E**–**G**):Transmission electron micrographs of leaf tissue of soybean treated with 150 mM NaCl and sprayed with TiO_2_ NPs; **(E)** The cell with many chloroplasts; (**F** and **G**) chloroplasts

After treatment with NaCl (150 mM), the major difference occurred in the chloroplasts. The TEM figures demonstrated that chloroplast shape and ultrastructure were altered by salt stress (Fig. 5A-C). An increase in the number of plastoglobules and the deformation of the membranes of the thylakoids were also observed when stressed by salt. Clearly, there was an enhancement when spraying with TiO_2_ as the chloroplasts were relatively larger than the salted plant and lying close to the cell wall, small number of plastoglobules, larger number of starch grains, grana and thylakoid lamellae are numerous and the cell contains relatively large vacuole (Fig. 5D-F).

#### Data inter-relationship analysis

**Fig. 6:**
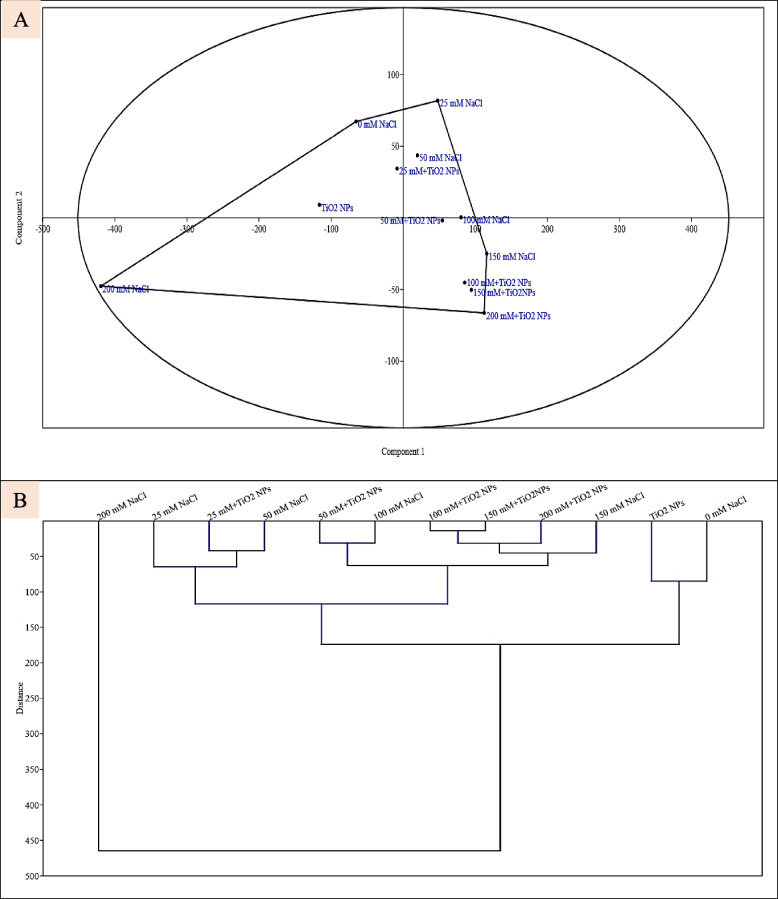
Data inter-relationship between different treatments (**A**) Principal component analysis (PCA) and (**B**) Hierarchical clustering analysis (HCA)

Both PCA and HCA displayed the relationships between different treatments (12 treatments) (Fig. 6A, B). This figure clearly confirmed the role of TiO_2_ NPs in lessening the detrimental effects of salinity on soybean plants.

## Discussion

The results indicated that salinity has a growth-inhibiting effect on soybean plants, and this effect is consistent with findings for cowpea, trigonella and wheat plants [45–47] respectively, suggesting that it may be a side consequence of Na ions. Salinity raises osmotic stress, which prevents the absorption and transportation of water. This inhibition triggers a series of events that can slow down the rate of stomatal opening, assimilation of CO_2_, and photosynthetic activity [48, 49]. Additionally, salt leads to nutritional imbalance, increased creation of ROS, suppression of enzymatic activity, interruption of cell division, and elongation, all of those having a detrimental effect on biological membranes and cellular components and reduce biomass production [50, 51].

Chlorophyll is one of the most significant biochemical characteristics that might indicate a plant’s state of health and its level is correlated with the availability of water and plant nutrients [52, 53]. Concerning the pigment fractions determined, under salt stress, soybean leaf total Chl. and pigment contents are significantly reduced with increasing NaCl levels as contrasted with the control. These results corroborate with Metwally and Soliman [7], Abdelhameed et al. [46] and Dief et al. [54] regarding the suppressing effects of salt stress on pigment contents of tomato, fenugreek and *T. aestivum* plants, respectively. It was thought that the sluggish synthesis or rapid degradation of these pigments in cells by enzymes is the cause of the decrease in these pigments under salt stress. For instance, the chlorophyllase enzyme causes the Chl. to degrade, which is detrimental to the photosynthetic process (Rady et al. [55]). Furthermore, our findings support the findings of Hameed et al. [56], which reported that salinity induces significant alterations in the structure and function of photosynthetic pigments by causing an excessive buildup of Na ions.

Most interestingly, the present investigation showed that TiO_2_ NPs increased the growth of soybean plants by rising their shoot and root fresh, dry weights, shoots height and root length under control and saline conditions. Also, TiO_2_ NPs application caused a significant enhancement in the total Chl and pigment fractions. The increased growth of shoots and roots may be linked to improved photosynthesis by the application of TiO_2_ NPs. Numerous researches stated that green synthesized TiO_2_ NPs enhance various photosynthetic pigments, resulting in increased growth and photosynthetic activity; Abdel Latef et al. [57] and Gohari et al. [58] described that TiO_2_ NPs increases plant agronomic parameters and photosynthetic pigments in plants exposed to salt. Where TiO_2_ NPs not only improve photosynthetic pigments and lower ROS generation, but they also slow down the pigments' breakdown [21]. Moreover, a study by Rahneshan et al. [59], using TiO_2_ NPs improved photosynthesis and lowered the detrimental impacts of salt while increasing the rate of macro- and micronutrient absorption. This finding is supported by the fact that TiO_2_ NPs stimulated genes mostly involved in photosynthetic metabolism in leaves of *Arabidopsis thaliana* treated with them, according to a recent genome-wide transcriptome research [60]. Furthermore, Ze et al. [61] demonstrated that TiO_2_ NPs may enhance chloroplast light absorption by upregulating genes associated with light harvesting complex II. Together, our results support TiO_2_ NPs' beneficial regulating role in the photosynthetic system.

As well reported for number of plants, salinity stress elevated ROS generation due to salt stress, which then damages membraneʼs protein and lipid and consequently increases electrolyte leakage (EL). The current study showed that in both NPs and non-NPs sprayed soybean plants, the EL of the leaves increased dramatically with increasing salinity stress. This supports the outcomes of Ahmad et al. [62, 63], where they demonstrated that salinity stress increased H_2_O_2_ in mustard with concomitant increase in membrane EL where increased H_2_O_2_ production causes disturbances in membrane and cellular homeostasis. Additionally, NaCl-induced ROS that react with membrane proteins cause peptide chain fragmentation and proteolysis, which compromise membrane stability. After treating both the shoot and the root with 300 mM NaCl for 21 days, Kumar et al. [64] reported that the MSI of the genotype that is vulnerable to salt decreased by approximately 45–50%. EL is inversely correlated with membrane stability. Clearly, TiO_2_ NPs increased the MSI and played a significant role in wheat plants’ water relations under salt stress [47] also, these NPs scavenge ROS, prevent a sustained oxidative burst and stabilize membranes to prevent EL [65]. It is worth stating, according to Hajizadeh et al. [66] that SiO_2_ NPs application at 50 mg L^−1^ cause a drop in EL and a boost in MSI were noted at 30 mM salinity.

In this study, rising salinity levels caused the production of protein and antioxidant enzymes (CAT, POX and APX) in soybean plants. As well, under salinity stress, the exogenous administration of TiO_2_ NPs considerably increased these values further. Our results are in line with those of Mustafa et al. [47], who applied TiO_2_ NPs to plants of *Triticum aestivum* L. that were stressed by salt and observed comparable outcomes. Under salt stress, protein increase may produce a storage form of N that can be stored and used later on, and it may also be essential for osmoregulation [67]. Kapoor and Srivastava [68] in a study on *Vigna mungo* have observed an increase in protein content with increasing salt concentration. Overall, the results corroborate those of Amini and Ehsanpour [69], who reported that the tomato plant (*Lycopersicon esculentum*) increased in protein content when treated with salt. Furthermore, the application of TiO_2_ NPs was shown to increase the protein content, which is in good agreement with the findings of Leopold et al. [70]. Increased protein concentration may result from soybean plants' more effective osmotic control system, which halts protein loss in response to salt stress [71]. This increase in proteins stabilizes the membrane and promotes plant growth in saltwater environments [72].

To withstand oxidative damage in harsh environments like salt stress, plants have evolved a system of antioxidant defence, which includes SOD, APX, POX and CAT enzymes [58]. At biochemical level, these enzymes are important antioxidant enzymes responsible for scavenging ROS because they effectively inhibit the build-up of O_2_^.−^ and H_2_O_2_ and reduce the harmful impact of ROS [73]. Consequently, they delay the oxidative stress that causes harmful effects on a number of delicate molecules, including lipids and proteins. Similar increase in CAT activity under salt stress has been stated in different plants as in common bean [74] and wheat [75]. Increased POX activity under salt stress has been detected in wheat plants [75, 76]. Our results concur with those of Weisany et al. [77], who reported that greater levels of oxidative stress led to an increase in the enzymatic activities of CAT and APX in soybeans under salt stress. Positive interactions may occur in relation to the increased antioxidant enzyme activities of soybean plants treated with TiO_2_ NPs under salt stress, which is expected to result in improved signalling towards the activation of these defence enzymes. These outcomes resemble those published by Gohari et al. [58]. The TiO_2_ NPs affected the ROS system of plants which was dependent on dose level, plants species and duration of exposure. For example, Abdel Latef et al. [57] demonstrated that 0·01% TiO_2_ NPs application boosted the enzymatic antioxidant defense in broad bean plants under salt stress. TiO_2_ NPs increased the SOD activities during germination in onion while CAT and POX activities varied with the doses applied [78]. This indicates that TiO_2_ NPs could alleviate the damage of ROS, where following the administration of TiO_2_ NPs, ROS detoxification may be the result of stabilized cell composition and enhanced membrane physical characteristics. According to Lei et al. [79], the use of TiO_2_ NPs under drought stress enhanced the activities of antioxidant enzymes in plants by lowering MDA and enhancing membrane integrity (less EL).

To understand the possible reasons for the observed changes in physiological and biochemical characteristics in soybean plants under different treatments, ultrastructural differences between control, TiO_2_ NPs and NaCl-treated leaf cells were studied. Cell structure has been linked to a number of alterations in response to environmental stimuli, water and mineral availability, and other factors. These changes are mainly in chloroplast structure, cell membrane, nucleus and other cell organelles. The ultrastructure abnormalities of cell organelles are indicative of metabolic disturbances caused by environmental stresses, especially salt stress. The plant species and its degree of salt tolerance determine how salinity affects the size, number, lamellar organisation, lipid and starch accumulation, and trafficking across the chloroplast membrane [56]. Chloroplast size variations would impact photosynthesis by altering the light-path within leaf cells [80]. The current findings shown that the number of chloroplasts decreases following treatment with 150 mM NaCl. Additionally, there are alterations to the chloroplast structure, such as changes to the lamellar organization that cause chloroplast shrinkage. and an unrecognizable grana structure. Moreover, the abundant plastoglobuli and large starch granules were observed. These changes are in good conformity with that of Blumenthal-Goldschmidt and Poljakoff-Mayber [81] and Papadakis et al. [82] under highly saline conditions.

Similarly, a study by Qiu et al. [83] revealed that the plants treated with 50% salinity showed significantly diminished stroma, grana and thylakoids in their chloroplasts; in some cases, they were even structurally distorted. Few grana thylakoids were compact and tightly packed; occasionally, they appeared to be merging. They were disorganised and included more plastoglobuli and starch granules. Certain plants, including Atriplex species, have the ability to deposit lipids in order to protect their cell structure from the damaging effects of salt-induced toxicity [84, 85]. Cucumber leaves have also been shown to exhibit stress-induced chloroplast envelope disintegration and an increase in plastoglobuli in thylakoid membranes [86]. Changes in glycophyte thylakoid membranes or swelling of the chloroplasts may be related to the ionic component of salt [56]. Early electron microscopic studies by Greenwood et al. [87] demonstrated the existence of osmiophilic globuli, or "plastoglobules," inside other plastids and chloroplasts. In general, they change in size and quantity during plastid development and differentiation and significantly increase under stressful circumstances concurrent with the breakdown of the thylakoid membrane. Additionally, the presence of plastoglobules proves the lipid release from thylakoid membranes and electron transportation disturbances [88].

In the interaction between TiO_2_ NPs and salt stress, soybean leaf maintains its structure by more increase in number of chloroplasts compared to salt treated leaf only. Also, the internal structure of the cell was restored and the damage was decreased. Notably, maintenance of greater growth, pigments, MSI while decreasing EL content under salt stress is one of the remarkable effects of TiO_2_ NPs. All these effects indicate the role of TiO_2_ NPs in maintaining the cell ultrastructure of soybean under salt stress. In the study of Younis et al. [89], pretreatment of wheat plants with Si NPs reduced the detrimental ultrastructural changes brought on by heat stress by keeping the nuclear envelope intact, the normal dispersion of chromatin, and chloroplast structure unaltered.

## Conclusion

In this extensive research, TiO_2_ NPs were green synthesized using *A. vera* aqueous extract and their potential in ameliorating tolerance in soybean plants against salinity had been studied through an in vivo pot experiment. Treatment of control and salt stressed soybean plants by TiO_2_ NPs (30 ppm) showed an improvement in the vegetative growth of soybean plants by increasing its pigment fractions, MSI and protein content. Also, there was a significant increment in enzymatic antioxidants (CAT, APX and POX) with TiO_2_ NPs application. Additionally, the detrimental effects of salt on soybean plants had lessened by lowering EL levels. Moreover, salt stressed soybean plants showed ultrastructural deformation which was lessened by TiO_2_ NPs application. Collectively, this study concluded the advantageous impact of TiO_2_ NPs in mitigating salinity stress in soybean plants. Despite these advantages, the use of TiO_2_ NPs in agriculture requires careful assessment due to potential environmental and health risks, such as NP accumulation in soil and water and their long-term effects on ecosystems. Further research and regulations are needed to optimize their use and ensure safety.

## Supplementary Information

Supplementary Material 1.

## Data Availability

All data has been provided with the manuscript.

## Abbreviations

MSI: Membrane stability index
EL: Electrolyte leakage
CAT: Catalase
APX: Ascorbate peroxidase
POX: Peroxidase
TiO_2_ NPs: Titanium dioxide nanoparticles
SOD: Superoxide dismutase
