# Supplementary material for: Efficacy of green synthesized titanium dioxide nanoparticles in attenuation salt stress in Glycine max plants: modulations in metabolic constituents and cell ultrastructure
Source: BMC Plant Biol. 2025 Feb 18;25:221. doi: 10.1186/s12870-025-06194-6 (PMC11834211; doi:10.1186/s12870-025-06194-6)
Supplement: Supplementary file 1 — Supplementary Material 1. [file 12870_2025_6194_MOESM1_ESM.docx]

| **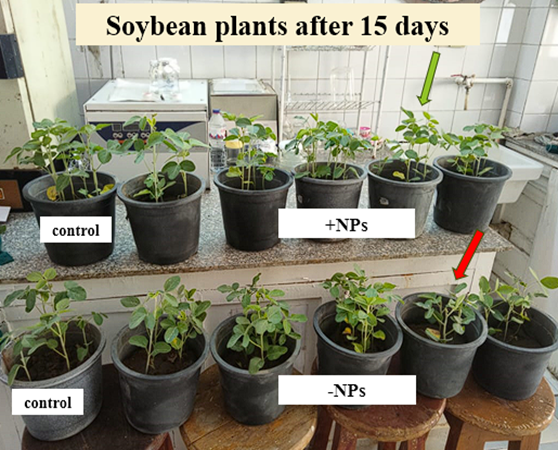**  **(A)** |
| --- |
| **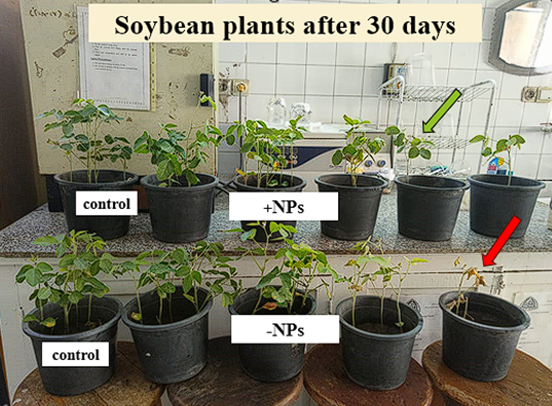**  **(B)** |

**Suppl. Fig. 1**: The growth response of TiO_2_ NPs (+NPs) and non-TiO_2_ NPs (**-**NPs) treated soybean plants grown under different NaCl concentrations after (**A**):15 and (**B**): 30 days from salt application.
